# Oxidative Stress and Thrombosis during Aging: The Roles of Oxidative Stress in RBCs in Venous Thrombosis

**DOI:** 10.3390/ijms21124259

**Published:** 2020-06-15

**Authors:** Qinhong Wang, Rahima Zennadi

**Affiliations:** 1 Division of Hematology, Department of Medicine, Duke University School of Medicine, Durham, NC 27710, USA; qinhong.wang@duke.edu; 2 Box 2615 Duke University Medical Center, Durham, NC 27710, USA

**Keywords:** VT/E, aging, red blood cell, oxidative stress, reactive oxygen species, antioxidant defenses, redox regulation, venous thrombosis

## Abstract

Mid-life stage adults are at higher risk of developing venous thrombosis (VT)/thromboembolism (VT/E). Aging is characterized by an overproduction of reactive oxygen species (ROS), which could evoke a series of physiological changes involved in thrombosis. Here, we focus on the critical role of ROS within the red blood cell (RBC) in initiating venous thrombosis during aging. Growing evidence has shifted our interest in the role of unjustifiably unvalued RBCs in blood coagulation. RBCs can be a major source of oxidative stress during aging, since RBC redox homeostasis is generally compromised due to the discrepancy between prooxidants and antioxidants. As a result, ROS accumulate within the RBC due to the constant endogenous hemoglobin (Hb) autoxidation and NADPH oxidase activation, and the uptake of extracellular ROS released by other cells in the circulation. The elevated RBC ROS level affects the RBC membrane structure and function, causing loss of membrane integrity, and decreased deformability. These changes impair RBC function in hemostasis and thrombosis, favoring a hypercoagulable state through enhanced RBC aggregation, RBC binding to endothelial cells affecting nitric oxide availability, RBC-induced platelet activation consequently modulating their activity, RBC interaction with and activation of coagulation factors, increased RBC phosphatidylserine exposure and release of microvesicles, accelerated aging and hemolysis. Thus, RBC oxidative stress during aging typifies an ultimate mechanism in system failure, which can affect major processes involved in the development of venous thrombosis in a variety of ways. The reevaluated concept of the critical role of RBC ROS in the activation of thrombotic events during aging will help identify potential targets for novel strategies to prevent/reduce the risk for VT/E or VT/E recurrences in mid-life stage adults.

## 1. Introduction

Aging contributes to an elevated incidence of venous thrombosis (VT)/thromboembolism (VT/E) [1,2], the third most common cause of cardiovascular death worldwide. It has been documented that the incidence of VT/E is two to seven times higher in patients above the age of 55 as compared to a younger cohort [3]. A large sum of first-time VT/E occurs in patients that are ≥ 45 years of age [4]. These epidemiological findings have sparked considerable interest in characterizing changes in the coagulation system as a function of aging, since in light of increasing life expectancy, VT/E will become a greater health care issue [5]. Hence, aging is not only an important biological issue, but also a crucial socioeconomic factor affecting an ever-increasing aging population. A normal coagulation pathway represents a balance between thrombosis and hemorrhage. This thrombo–hemorrhagic balance is maintained in the body by intricate interactions between coagulation and the fibrinolytic system, as well as platelets and vessel walls. Usually, the coagulation process is under the inhibitory control of several inhibitors that limit clot formation, thereby avoiding thrombus propagation. This delicate balance is interrupted whenever the procoagulant activity of the coagulation factors is increased, or the activity of naturally occurring inhibitors is decreased. The process of aging can disturb this delicate balance, promoting venous thrombosis. Aging is associated with overproduction of reactive oxygen species (ROS), which could evoke a series of physiological changes that create a discrepancy between thrombosis and hemorrhage [6,7,8,9]. These pathophysiologic changes involve anomalies in blood coagulability, including vessel function [10,11], blood flow, and the coagulation pathways [12]. Understanding how aging and the associated oxidative stress disrupt the coagulation cascade to initiate venous thrombosis could help in the design of more effective therapies to reduce the risk of VT/E, and/or VT/E occurrences in mid-life stage humans. Venous thrombi or clots have high RBC and fibrin content, which makes them distinct in appearance, and since this observation has been made, red blood cells (RBCs) are now accepted as a critical mediator of venous thrombosis. In this review, we will mainly focus on the valuable role of the RBC in oxidative stress-related thrombotic processes, and how oxidative stress within the RBC affects RBC quality and function, which contribute to the development of thrombosis during aging. The summarized information will highlight useful knowledge of the role of oxidative stress in RBCs in precipitating thrombotic events in relation to aging.

## 2. Oxidative Stress Contribution to Venous Thrombosis during Aging

There is increasing evidence suggesting that aging is associated with an imbalance between oxidative stress and antioxidant status. Up-regulation of ROS-producing enzymes, such as nicotinamide adenine dinucleotide phosphate (NADPH) oxidase and myeloperoxidase, along with down-regulation of antioxidant enzymes, such as superoxide dismutase (SOD) and glutathione peroxidase (GPx), occur during aging. This imbalance may predispose one to thrombosis by impairing RBC quality and function, eliciting endothelial dysfunction, and activating platelets and leukocytes, consequently affecting the clotting system. Excessive ROS generation or a defect in the antioxidant defense system impacts a wide variety of biological molecules, lipids in the plasma and mitochondrial membranes, causing lipid peroxidation that impairs membrane selective permeability, proteins, resulting in structural instability and damage to their enzymatic function, and nucleic acids, thus inducing pathways of apoptosis. The endothelial cell lining is essential in triggering the prothrombotic events; an intact endothelial cell lining prevents platelets from adhering to the endothelium, and thus prevents platelet activation. However, an imbalance between the generation of ROS and antioxidant systems, as a function of aging, causes endothelial dysfunction and damage to endothelial cell lining. It has been postulated that the pathologic process of thrombosis begins with endothelial injury, and subsequently depends largely on a function of platelets, coagulation factors, and antithrombotic and fibrinolytic systems. Age-related endothelial dysfunction involves upregulation of the NADPH oxidase (NOX)- and cyclooxygenases (COXs)-dependent oxidative stress pathways [13], and overexpression of the antioxidant enzyme glutathione peroxidase 1 (GPX-1) protects from age-dependent increased venous thrombosis [14]. Oxidative stress and enhanced ROS production down-regulate the protective nitric oxide (NO) pathway, since reducing oxidative stress by the antioxidant vitamin C restores NO availability [15]. As a result of low NO availability, endothelium-dependent vasodilation is impaired [15], which leads to abnormal RBC adhesion [16], and may contribute to increased platelet activation [17]. Indeed, the aged blood vessel expresses less endothelial NO synthase (eNOS) [18], producing less NO [15]. Investigators have shown that aortas from old and middle-aged rats produce much less NO in response to the calcium ionophore A23187 than aortas from young rats do [19]. It seems that the decline in vascular NO production may be a characteristic feature of mammalian aging. Impaired NO availability can be caused by inactivation of NO synthase (NOS), since the aged blood vessel expresses less eNOS [18], and the levels of an endogenous inhibitor of NOS, asymmetric dimethylarginine (ADMA), are increased in healthy elderly subjects [20]. NO *S*-nitrosylates the active site, cysteine residues, of several caspases, predominantly caspase-3, the crucial mediator of apoptosis [21,22,23]. Endothelial apoptosis can trigger development of thrombosis [24]. Furthermore, because of its potent procoagulant activity, tissue factor (TF) gene expression and activity are tightly regulated to maintain hemostasis while preventing thrombosis. Endothelial injury-associated oxidative stress promotes TF expression, which can be regulated by NO [25,26]. Exposure of TF-expressing cells during injury allows the complex formation of TF with factor (F) VII. TF and FVII form an equimolar complex in the presence of calcium ions (Ca^2+^), leading to the activation of FVII on a membrane surface, and initiation of the coagulation cascade to generate thrombin. Oxidative stress-associated endothelial injury may be triggered by elevated ROS levels in RBCs during aging, since the RBC is a major source of ROS production and oxidative stress.

## 3. RBC Oxidative Stress and Its Effect on RBC Quality during Aging

The main function of RBCs is to transport oxygen from the lungs to tissues via the oxygen-transport protein hemoglobin (Hb), which is enriched in iron (Fe^2+^). Hb within the RBC is protected from oxidative aggressions by enzymatic and small molecule antioxidants, as well as the RBC membrane, which provides a physical barrier against oxidation by exogenous sources of ROS in the circulation that can damage the RBC and hamper its function. Importantly, endogenous ROS are continuously generated by the slow autoxidation of oxygen-carrying Fe^2+^ containing Hb (HbFe^2+^O_2_) to ferric (Fe^3+^)-containing methemoglobin (HbFe^3+^) (that can no longer carry oxygen) [27]. Reduction to the oxygen-carrying ferrous (HbFe^2+^O_2_) form occurs rapidly, in order to restore oxygen capability and prevent cellular injury, which may be triggered by oxidation intermediates carrying. The spontaneous oxidation of Hb is the initial oxidative process in the single electron oxidation of ferrous to ferric Hb, with the production of superoxide (O_2_^•−^) that rapidly dismutates to form hydrogen peroxide (H_2_O_2_) (Equation (1)). Another important oxidative pathway is the reaction between H_2_O_2_ and both ferrous (Equation (2)) and ferric Hb (Equation (3)), resulting in heme degradation and the release of free iron [28]. These reactions lead to the generation of the potent oxidizing ferrylHb (HbFe^4+^) species, as well as secondary radicals from the reaction between H_2_O_2_ and either oxyHb or methemoglobin (metHb). This pathway has also been proposed to be important in mediating hemolytic injury [29]. Furthermore, RBC membrane-bound NOX enzymes contribute to the production of endogenous ROS [30]. Additionally to transporting and delivering O_2_, RBCs act as hypoxia sensors that release bioactive nitric oxide (NO) derivatives, leading to NO-dependent vasodilation and increased blood flow to meet tissue oxygen demand [31]. This occurs after transfer of NO from heme iron (iron nitrosyl, FeNO) to cysteine thiol (forming S-nitrosothiol, SNO). However, the O_2_-carrying ferrous (HbFe^2+^O_2_) form is able to react with NO to generate ferric Hb and nitrate (Equation (4)).

HbFe^2+^O_2_ → HbFe^3+^ + O_2_^•−^ (1)

HbFe^2+^O_2_ + H_2_O_2_ → HbFe^4+^ + O^2−^ + H_2_O + O_2_ (2)

HbFe^3+^ + H_2_O_2_ → Hb^•+^Fe^4+^ + O^2−^ + H_2_O (3)

HbFe^2+^O_2_ + NO → HbFe^3+^ + NO_3_ (4)

**Figure 1:**
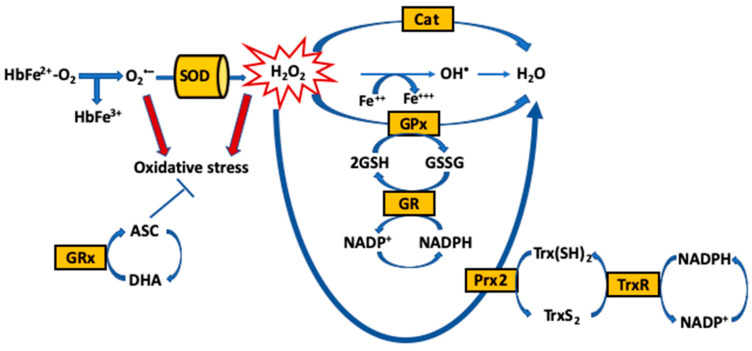
Redox hemostasis in RBCs. Heme oxidation of oxyHb (autoxidation of Hb) to methemoglobin (metHb) generates superoxide anions (O_2_^•−^). Superoxide anions are scavenged by superoxide dismutase (SOD) enzymes and transformed into hydrogen peroxide (H_2_O_2_). Three main pathways exist in RBCs to convert hydrogen peroxide to water (H_2_O). Nicotinamide adenine dinucleotide phosphate (NADPH)/glutathione (GSH)-dependent pathway: NADPH is used as a substrate for glutathione reductase (GR) to recycle glutathione disulfide (GSSG), the oxidized form, back to reduced GSH. GSH is then utilized by two enzymes: first, glutathione peroxidase (GPx) for the direct breakdown of hydrogen peroxide, and second, glutaredoxin (GRx) to convert ascorbate (ASC) to Dehydroascorbic acid (DHA) by the plasma membrane redox system and diffusing reactive oxygen species (ROS). NADPH/thioredoxin (Trx)-dependent pathway: NADPH is also used as a substrate by the thioredoxin reductase (TrxR), which keeps the cofactor Trx in the reduced state (Trx(SH)2). Trx(SH)2 serves as an electron-delivering system to the membrane-associated peroxiredoxin 2 (Prx2), which scavenges hydrogen peroxide, and Trx(SH)2 is then oxidized, forming disulfide bridges (TrxS2). NADPH-independent pathway: Hydrogen peroxide is broken down into H_2_O via pathway 3 involving catalase (Cat).

The maintenance of redox homeostasis is essential for cell survival [32,33]. Yet, RBCs are highly susceptible to ROS that oxidatively damage the macromolecules, and ultimately lead to impaired oxygen delivery, aging and cell death [32]. To neutralize ROS in access and the resultant oxidative stress, RBCs have an extensive antioxidant system, comprised of enzymatic antioxidants, including catalase (Cat), SOD, GPx, glutathione reductase (GR) and peroxiredoxin-2 (Prx-2) [33,34,35,36], and non-enzymatic low-molecular weight antioxidants (reduced and oxidized), either produced intracellularly [glutathione (GSH)/glutathione disulfide (GSSG) and NADH/NADPH] or up-taken by the cells [α-tocopherol (Vitamin E), ascorbate (ASC), bioflavinoids and selenium] [37] (Figure 1).

During the progression of aging, it has been well documented that the redox homeostasis is generally compromised due to an imbalance between prooxidant and antioxidant biomarkers [38,39]. In aging, elevated concentrations of H_2_O_2_ and organic hydroperoxides, and decreased GSH/GSSG ratio and glutathione S-transferase activity, have been detected in RBCs [40]. Studies have also shown age-dependent alterations in RBC malondialdehyde (MDA), intracellular GSH and membrane sulphydryl (SH) groups in the older Indian population, and decreased GPx activity [41,42]. The absence of protein synthesis machinery, due to enucleation during RBC maturation, renders these cells even more vulnerable towards oxidative stress-mediated damage [43]. Therefore, during aging, the ability of the antioxidant system to neutralize the endogenous ROS in access can be limited, and the un-neutralized ROS in the RBC can damage the RBC membrane, which not only dampens oxygen delivery to the tissues, but also impairs hemorheological properties, thus reducing the flow of RBCs through the microcirculation [44]. As a result, RBCs travel in close proximity, or even make contact with the vasculature [44,45], up-taking in addition ROS released from neutrophils [46], macrophages and endothelial cells [47,48]. The age-associated excessive ROS accumulation mainly deteriorates cellular structural and functional activities, with subsequent ROS-mediated accrual of macromolecules [49]. Disruption of the organizational cell structure during oxidative stress exemplifies an ultimate mechanism in system failure [37].

The ROS generated on the RBC membrane through Hb autoxidation are ideally located to react with membrane lipids and proteins, producing lipid peroxidation, and modified membrane proteins, altering conformation of cytoskeleton proteins [32,50,51,52,53]. Several studies have evidenced increased lipid peroxidation levels in rat erythrocytes and other tissues with the advancing of age [54,55]. Erythrocyte band 3-associated enzymes, such as phosphofructokinase and glyceraldehydes-3-phosphate dehydrogenase, are also affected by the accumulation of lipid peroxidation [56]. Partial degradation of band 3 can, in addition, be observed in RBCs due to oxidative reactions-mediated activation of caspase 3 [57,58], affecting the interactions of band 3 with cytosolic proteins, as well as the linkage to ankyrin and the cytoskeleton, resulting in phosphatidylserine (PS) exposure, a negatively charged phospholipid normally present on the cytoplasmic side [59]. This dramatic rearrangement of the membrane has been shown to involve a concomitant decrease in deformability. Likewise, oxidative stress can inhibit calcium (Ca^2+^) ATPase, an enzyme regulating the intracellular concentration of Ca^2+^ [43,60]. Enhanced intracellular Ca^2+^ activates the Gardos channel, and leakage of potassium from the RBC, thus affecting cation homeostasis-induced shrinkage of the cell and impaired deformability [61,62]. Additionally, aging is related with a decline in GSH in RBCs, which results from reduced expression of the proteins involved in GSH synthesis and its reduction [63]. Reduced GSH, an intracellular non-enzymatic and thiol-containing antioxidant compound, has also been described to affect free radicals scavenging and the protection of proteins and lipids in the membrane from free radical-mediated oxidation [42,64]. Reduced erythrocyte acetyl cholinesterase (AChE) activity, which is susceptible to oxidative stress-mediated dysfunction, was also evidenced in aged humans [65,66], suggesting that reduction in AChE activity might be caused by excessive oxidative damage during aging. Previously, AChE in RBCs was evidenced as a biomarker of membrane integrity [67]. Depending on the degree of endothelium integrity, the plasma acetylcholine (ACh) induces vasodilation or vasoconstriction, through the amount of NO synthesized by endothelial cells and released to the vessels [68,69]. Furthermore, aging is associated with a defective autophagy process, which plays a very important role during red cell maturation. The autophagy process is known to be active at a basal level at a young age, while in old age this process becomes impeded and contributes to the increased oxidative stress, the accumulation of damaged cell organelles, and oxidative stress-mediated protein aggregation, possibly contributing to the development of several age-dependent diseases [70,71]. Loss of autophagy in erythroid cells leads to a defect in mitochondria removal and severe anemia in vivo [72]. Normal aging is therefore characterized by the slow buildup of deleterious products, such as oxidized proteins, and advanced glycation and lipoxidation of end products in RBCs due to increased oxidative stress. Age-dependent vulnerability of RBCs towards oxidative stress, as well as increased levels of oxidative stress biomarkers in these cells, affects membrane structure and function, causing loss of membrane integrity, decreased deformability, accelerated aging, and probably hemolysis.

Aged and apoptotic RBCs are normally cleared from the circulation by macrophages. Immunologically silent phagocytosis of aged and apoptotic RBCs is critical to maintaining RBC homeostasis and innate immune balance [73,74]. Phagocytosis of these defective cells or cellular debris triggers immunosuppressive signaling, with the release of anti-inflammatory cytokines, leading to peripheral immune tolerance. Approximately 2.4 million defective RBCs are phagocytosed per second by Kupffer cells, the macrophages in the liver, with new erythrocytes produced at the same rate [75]. Without recycling, iron deficiency would occur due to the rapid synthesis of new hemoglobin. Given that at least 300 billion cells undergo apoptosis every day in our body [75], nutrient recycling via phagocytosis is crucial for tissue renewal. Aging reduces phagocytosis of apoptotic cells both in vitro and in vivo. Dendritic cells from elderly subjects showed a more reduced capacity to phagocytose apoptotic cells or Dextran in vitro than the dendritic cells from young subjects [76]. Similarly, peritoneal macrophages in aged mice exhibited reduced phagocytic clearance of apoptotic Jurkat cells [77]. As a result, these aged and defective RBCs, and likely free Hb, heme, heme-loaded vesicles and iron, can accumulate in the circulation.

## 4. Oxidative Stress in RBCs and Its Effect on Venous Thrombosis during Aging

**Figure 2:**
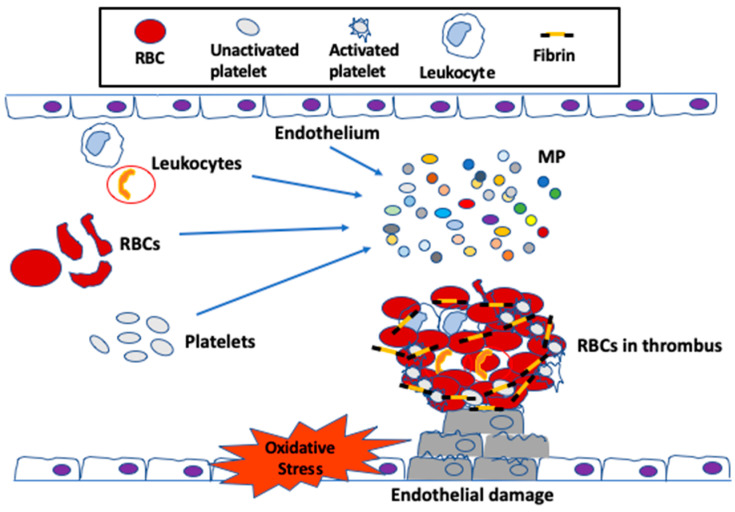
RBC contribution to venous thrombus formation. In normal conditions, erythrocytes travel in the center of blood flow and platelets travel closer to the endothelial cells. When the endothelium damage occurs by oxidative stress, RBCs can adhere to the injury-exposed sub-endothelial matrix prior to the arrival of platelets and leukocytes. RBCs may contribute to thrombin generation within thrombi. Once incorporated into venous thrombi, RBCs increase thrombus size, and reduce thrombus permeability and susceptibility to lysis. Oxidative stress can also cause endothelial-, RBC-, platelet- and leukocyte-derived microparticles (MPs), which may also adhere to the endothelium or extracellular matrix, activate platelets and other cells, and enhance local thrombin generation during thrombosis. MPs are small membrane vesicles, which play an important role in coagulation. RBC- and platelet-derived MPs, on the other hand, can also initiate thrombin generation. After formation of the fibrin plaque, RBCs become intertwined within the thrombus to stabilize and strengthen its structure.

Until recently, RBCs have been always considered as bystander in hemostasis and thrombosis. During coagulation, thrombin cleaves N-terminal peptides from the Aα- and Bβ-chains of fibrinogen, which is comprised of two pairs each of Aα, Bβ and γ chains, arranged as a rod-like protein, promoting the formation of protofibrils and, subsequently, the network of insoluble fibrin fibers. The fibrin network is stabilized by the enzyme factor XIIIa, which cross-links γ-γ and γ-α chains within the network. The fibrin network serves as scaffold for the binding of endothelial cells, leukocytes, platelets and plasma protein to the clot. RBCs were thought to be simply trapped in the fibrin network or mesh. The first published clinical findings have shown that RBC transfusion significantly improved bleeding times in thrombocytopenic patients, whose platelet counts remained low, suggesting a role for RBCs in blood coagulation [78]. Since then, the effect of RBCs on thrombosis has been gradually valued, and these enucleated cells are now recognized as essential players in promoting venous thrombosis and enhancing thrombus stability [79,80,81]. RBCs support the activation of the coagulation factor cascade, and these cells are incorporated into thrombi via specific interactions [82,83,84]. For instance, RBCs interact not only with plasma proteins, most notably fibrinogen [85], but also with activated endothelial cells [80]; this RBC–endothelial cell interaction was demonstrated in a study of arterial thrombosis, describing that RBCs were the first cells to adhere to FeCl_2_-treated intact endothelium [80], prior to the arrival of platelets [84]. Increased RBC–endothelial cell and RBC–platelet interactions impact blood viscosity, which leads to activation of the coagulation system, subsequently increasing the risk for thrombosis and vascular complications (Figure 2).

**Figure 3:**
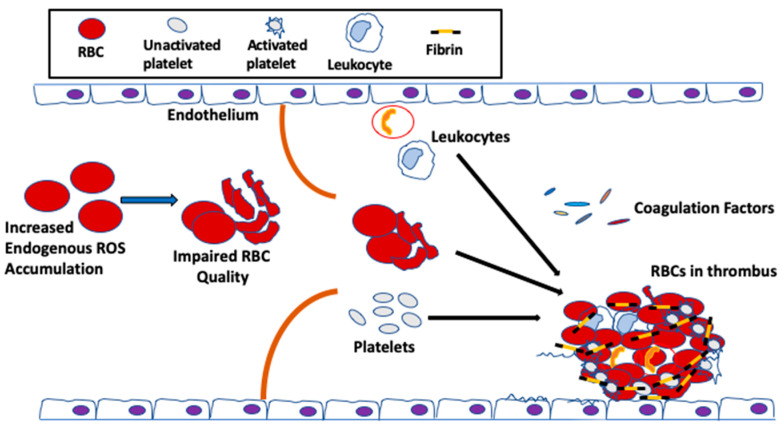
Potential contributions of RBCs to venous thrombus formation during aging. During aging, increased ROS accumulation in RBCs affects membrane structure and function, causing loss of membrane integrity, decreased deformability and accelerated aging, thus impairing RBC quality. As a result, the flow of RBC through the microcirculation is reduced. Venous thrombi form slowly in stasis or low flow and are RBC- and fibrin-rich. In veins, aged RBCs aggregate into stacked rouleaux structures, increasing blood viscosity. These abnormal RBCs can also directly or indirectly adhere to the vessel wall and may contribute to thrombin generation within thrombi. Once incorporated into venous thrombi, RBCs increase thrombus size and reduce thrombus permeability and susceptibility to lysis. During aging, abnormal RBCs and RBC-derived microvesicles may also adhere to the endothelium or extracellular matrix, activate platelets and other cells, and enhance local thrombin generation during thrombosis.

It has been postulated that RBC abnormalities, such as loss of regular phospholipid asymmetry, can predispose a patient to thrombosis [86,87,88], even when all other clotting factors, including the coagulation factors, the vascular endothelium, leukocytes and platelets, are normal. Indeed, clinical and epidemiological studies have implicated RBC quality in venous thrombosis, promoting clot formation and increasing clot stability [87,88,89]. Similarly, nonerythroid diseases, such as diabetes mellitus, that indirectly alter RBC properties can also result in an elevated clotting potentials [90,91,92]. During aging, the quality of the RBC in the circulation is negatively affected by excessive oxidative stress, caused by not only elevated endogenous ROS produced from Hb autoxidation, but also extracellular ROS [93,94], released by endothelial cells [95], neutrophils and macrophages [48,96], and up-taken by these enucleated cells, as discussed above. Thus, because the ability of the antioxidant system to neutralize ROS is limited during aging [42], the vulnerability of the RBCs towards oxidative stress-mediated damage is exacerbated. As a result, and as discussed earlier, the un-neutralized ROS in the RBC affect the membrane structure [32], which could reduce the flow of RBCs through the circulation, thus impairing RBC function in hemostasis and thrombosis [43] (Figure 3).

It has been reported that increased ROS production within the RBCs induces PS exposure on the cell surface, and impacts RBC adhesive function [97]. Undeniably, reducing RBC ROS generation with manganese (Mn) porphyrins, which are found to suppress RBC NOX activity [97], improved eryptosis, reflected by reduced RBC PS exposure in a mouse model of sickle cell disease, suggesting that NOX-dependent ROS, produced in excess within the RBCs, can up-regulate PS exposure on the surface of the sickle cells [98]. It is therefore possible that excessive ROS build-up in RBCs during aging may directly contribute to PS externalization on the surface of these cells as well [99]. Increased NOX-dependent ROS production within the RBCs can mediate adhesion of these sickle cells to the vascular endothelium, both in vitro and in vivo [97]. PS exposed on the surface of the RBC membrane has been evidenced to contribute to endothelial adhesion of sickle RBCs [100]. Importantly, PS exposure on RBCs plays a critical role in the progression of vascular thrombosis [101,102,103,104,105]. It has been postulated that even a small fraction of RBCs exposing PS can contribute to thrombin generation, and this subpopulation of RBCs might explain about 30–40% of the thrombin-generating potential of whole blood [106], revealing the critical role of PS exposure in thrombosis development [107]. Studies have also found correlations between the levels of PS externalized on the RBC membrane and plasma levels of hemostatic markers, including prothrombin fragment F1.2, the thrombin–antithrombin complex, the plasmin–antiplasmin complex, and D-dimer, suggesting that increased PS exposure may cause a pathologic RBC procoagulant phenotype, a factor that can induce a hypercoagulable state [107]. Induction of PS externalization in RBCs requires elevated intracellular Ca^2+^, which activates scramblase, a molecule responsible for the translocation of PS between the two monolayers of a lipid bilayer of the cell membrane [108,109]. Intracellular ROS stimulates several Ca^2+^ transporters localized in the cell membrane, leading to extensive accumulation of Ca^2+^ within the cell, and vice versa [110,111], suggesting that oxidative stress is important for PS exposure on the RBC membrane. For a long time, the physiological function of Ca^2+^ in RBCs was obscure, and was believed to be limited to the involvement in RBC aging and clearance [112,113,114]. However, the Ca^2+^ in RBCs has crucial physiological functions, regulating a broad range of processes, including O_2_ transport [115], rheology [116], and clotting via the altering of the rheological properties of RBCs by provoking increased Ca^2+^ levels, or RBC aggregation [117,118]. Thus, aberrant Ca^2+^ homeostasis in RBCs results in increased risk for developing not only VT/E, but also severe, life-threatening systemic pathologies.

In addition, RBCs generate microscopic extracellular membrane structures named microvesicles (MVs) or microparticles (MPs). Most RBC-derived MPs expose PS [119]. Membrane microvesiculation is a physiologic process for mature RBCs. This process represents a well-regulated mechanism [120], and may contribute to irreversible membrane-carrying hemoglobin loss, or the exocytosis of damaged cell components in RBCs [119]. MPs are involved in various biological functions, such as thrombosis and hemostasis [121,122], and inflammation [123]. The best studies describing the relationship between MPs production and thrombosis are on hematological diseases [124]. MPs are involved in clinical situations characterized by hemolysis or endothelial activation. In sickle cell disease, the abnormal hemoglobin S autoxidation is involved in membrane instability, and favors MP shedding [125]. In this disease, the number of MPs correlates with the rate of intravascular hemolysis and the degree of coagulation activation [126,127]. PS-positive MPs potentiate thrombin generation [126,127,128] via FXIIa [122,126,129] while other studies have identified activated FXII as being the main factor in the coagulation cascade, possibly via a PS-mediated mechanism [130]. RBC-derived MPs could be considered a potential target for treatment of hemostatic disorders given their broad procoagulant activity [130]. Excess ROS-induced PS exposure on RBCs may accelerate the generation of MPs, promoting prothrombotic events during aging.

Inferior RBC quality-related aging may result in an increase in blood viscosity, which potentially reduces blood flow [131,132]. This rheological behavior caused by the RBCs, is one of the primary factors precipitating thrombotic processes [90,132]. Reduction in local flow allows RBCs to accumulate and cause a decrease in the wall shear stress, which lowers NO release [133,134]. Because NO prevents the activation of endothelial cells and platelets, a deficiency in NO promotes the interactions of platelets with the vascular endothelium and/or injury-exposed sub-endothelial matrix [16,135,136]. Reduced blood flow or blood stasis also allows RBCs to interact with themselves, forming RBC aggregates [137], and consequently triggering vein thrombosis [138]. RBCs also physically control platelet hemostasis [139], supporting shear-induced platelet adhesion largely by enhancement of platelet transport from the bulk flow to the bounding surfaces [140]. In the presence of RBCs, platelets are activated, leading to platelet FasL exposure, which activates FasR on RBCs that is responsible for externalization of PS on the RBC membrane, and subsequently accelerating thrombin generation [141]. RBC contact with platelets also enhances activation of α_IIb_β3 integrin receptor and expression of P-selectin on platelets, which intensify platelet aggregability and mediate increased platelet recruitment [142]. Platelet aggregation and degranulation are triggered by ATP and ADP released by RBCs in response to chemical and physical mechanisms [143]. With increasing age, higher levels of beta-thromboglobulin (β-TG) and platelet factor 4 (PF4) were documented [144]. A correlation exists between both ADP-induced aggregation and β-TG, and age in healthy volunteers [145]. Platelet aggregation can also be induced by RBCs through the release of hemoglobin, which lowers NO bioavailability [146,147]. Platelets activated by RBCs accelerate thrombin generation by providing a major site for assembly of the tenase (FIXa and FVIIIa) and prothrombinase (FXa and FVa) complexes [148], and also by directly participating in thrombus formation. In addition to platelets, defective RBCs could also adhere to the endothelium, interact with leukocytes, or aggregate with other cells to form platelet–leukocyte–RBC–endothelium; these adhesive and functional interactions play a significant role in thrombosis [80,97,149,150,151].

Another possible role for the RBCs in thrombosis during aging is the activation of the complement system by these cells. As a major constituent part of the innate immune system, the complement system not only connects innate with adaptive arms of the immune system, but also links the coagulation system with the immune system, a cross-talk vital for maintaining homeostasis [152]. It has been shown that heme-loaded, RBC-derived MVs activate the innate immune complement system, and cause an inflammatory reaction, leading to the cleavage of complements C3 and C5, the release of anaphylatoxins C3a and C5a, respectively, and the formation of the membrane attack complex C5b-9 [153], which stimulates procoagulant activity through platelet prothrombinase [154,155]. The terminal complement complex C5b-9 promotes the release of platelet factor V and the assembly of the prothrombinase complex, thereby potentiating the effects of thrombin on the activation of prothrombinase [154]. C5a could up-regulate TF expression by endothelial cells and neutrophils [156,157], promoting the extrinsic coagulation pathway. Clinical findings have shown significantly elevated markers of complement activation in sera of patients with sickle cell disease, as well as increased levels of surface-bound C3 fragments on sickle RBCs [158], suggesting a possible role for abnormal RBCs in the activation of complement system-mediated thrombosis [159]. RBC MPs can be released into the bloodstream due to intravascular hemolysis [122,160]. These RBC-derived MVs are a pathologically relevant form of heme carrier. The heme-loaded MVs activate the alternative and terminal complement pathway, which is initiated on the endothelial surfaces, resulting in activation of the thrombotic cascade [121,157,160]. Hemoglobin, on the other hand, triggers rapid P-selectin, C3aR and C5aR expression, and the down-regulation of CD46 on endothelial cells; processes associated with inflammation and organ injury, subsequently promoting blood coagulation [161]. Studies have also reported that RBC-derived MPs affect thrombin generation due to the presence of exposed PS on their membrane [129].

Moreover, advancing age is often associated with an altered hemostatic factor profile, typified by heightened plasma levels of hemostatic factors such as fibrinogen and coagulation FVII [162,163]. RBCs can bind to fibrinogen via an integrin receptor on the RBC membrane, either a β3 integrin, CD47, or both, promoting RBC aggregation [85,164,165]. It is also suggested that RBC membranes can activate FIX, which may serve as a triggering mechanism for blood coagulation [166]. It is therefore likely that age-associated oxidative stress in RBCs, combined with a “prothrombotic” hemostatic profile, accelerates thrombotic events, thus increasing the risk of VT/E.

## 5. RBCs as a Major Source of Oxidative Stress-Associated Thrombosis during Aging

Increased oxidative stress and ROS accumulation in RBCs during aging may induce RBC hemolysis. Following hemolysis, free Hb and heme can be rendered relatively inactive by the plasma proteins haptoglobin and hemopexin [167,168,169], and delivered safely to macrophages for phagocytosis [170,171]. However, oxidized Hb exhibits impaired plasma clearance, due to their low affinity for haptoglobin protein. Oxidation of Hb to ferri- and ferrylhemoglobin enables the release of heme and iron in proximity to neighboring tissues [172]. Free redox-active heme translocates rapidly into the endothelial cells, and initially triggers H_2_O_2_-mediated endothelial damage, but later, cyto-resistance to heme-induced damage arises by engaging the cyto-protective agents, heme oxygenase-1 (HO-1) and ferritin [173]. Endothelial cell toxicity occurs when the extracellular and intracellular defenses against oxidative stress are overwhelmed. 

The progressive release of redox-active iron and heme into the blood-stream triggers a chain reaction that is toxic to the vasculature, contributing to the development of vascular diseases [174]. Recent studies have suggested that free hemoglobin and heme stimulate the nuclear factor κB (NF-κB) under the control of a Toll-like receptor (TLR)-signaling pathway, involving TLR4 [175,176,177,178,179]. Hemoglobin-induced NF-κB activation regulates hypoxia inducible factor (HIF)-1α and HIF-2α [180]. These two transcription factors, once activated, cooperate to trigger chronic inflammation, vasoconstriction and endothelial permeability [180,181]. The induction of the antioxidant HO-1 can offset this chain reaction and may protect against tissue injury, consequently reducing the risk of recurrent venous thrombosis. Studies support this notion by showing that HO-1 deficiency in mice impairs thrombus resolution and exaggerates the inflammatory response to thrombus formation [182]. These studies are in accordance with other studies, demonstrating that long GT-repeat alleles in the HO-1 gene (HMOX1) are associated with decreased HO-1 anticoagulant activity, and hence an increased risk of thrombosis [183]. During aging, it has been documented that enhanced oxidative stress, caused by free iron and heme, is accompanied by compensatory induction of the antioxidant enzyme HO-1, which occurs through activation of the NF-κB pathway [184]. Effectively, HO-1 overexpression contributes to the pathological iron deposition and mitochondrial damage in brain aging and neurodegenerative disorders [185], suggesting that RBC oxidative stress-dependent hemolysis occurs during aging. Alternatively, studies have shown that oxidative stress-induced activation/phosphorylation of PECAM-1 [186] downregulates HO-1 via the master antioxidant transcription factor NF-E2-related factor-2 (Nrf2), and modifies intracellular ROS levels in human endothelial cells [187].

Cell-free hemoglobin can impact the bioavailability of NO, resulting in endothelial dysfunction and vasoconstriction [188]. Scavenging NO by plasma hemoglobin reduces NO availability, which alters blood cell adhesive function [189]. Extracellular heme derived from lysed erythrocytes can also induce NETosis, comprised of decondensed chromatin and DNA from activated neutrophils, causing endothelial activation and damage [190]. The disrupted and activated endothelial barrier can expose or release prothrombotic proteins, such as collagen, TF and von Willebrand Factor (vWF) [191,192], and chemotactic proteins, such as cytokines and surface adhesion molecules [193], into the blood, which then support further coagulation, platelet aggregation and leukocyte recruitment. Neutrophil extracellular traps (NETs) provide a scaffold and stimulus for thrombus formation. NETs perfused with blood cause platelet adhesion, activation and aggregation, erythrocyte recruitment, and fibrin deposition [194].

Free Hb can also bind to glycoprotein-1b alpha (GPIbα) on platelets, leading to platelet activation and binding to vWF, subsequently promoting thrombus formation. Hemoglobin–GP1bα interaction stimulates events such as platelet shape change, granule secretion and the inside-out signaling process, leading to activation of the ligand-binding function of integrin GPIIbIIIa [195]. The Lyn/PI3K/Akt/NO/cGMP/PKG/MAPK pathway reportedly plays an important role in GPIbα-mediated platelet activation and aggregation [195,196]. The activation of this pathway in platelets is generally seen along with other platelet receptor–ligand interactions, such as GP1bαIX–vWF and GPVI–collagen [197,198]. In addition to GP1bα, heme can induce platelet activation through C-type lectin like receptor-2 (CLEC-2) [199]. Heme up-regulates and binds to TF on macrophages as well, promoting TF-dependent coagulation activation [200]. Further, the TF pathway inhibitor (TFPI), the only physiologic regulator of TF activity, can be inhibited by oxidative stress, and exert a procoagulant effect [201].

As a result of increased ROS levels in the circulation, ROS may further favor a procoagulant state through the oxidative modification of proteins involved in coagulation. To mention a few mechanisms exerted by ROS, ROS can directly inactivate major anticoagulant proteins, protein C [202] and its upstream agonist thrombomodulin [203]. ROS can exert a prothrombotic role by oxidizing fibrinogen, thus accelerating the conversion to fibrin [204], and decrease thrombin binding to anticoagulants, the antithrombin III–heparin complex and thrombomodulin [205]. ROS can additionally reduce the heparin-binding capability of antithrombin [206], shed P-selectin, the circulating levels of which are associated with an increased risk of venous thromboembolism [207] and directly act as chemo-attractants for neutrophils [208].

## 6. Genetic Risk Factors and Venous Thrombosis

Venous thrombosis is associated with genetic risk factors [209]. Studies of venous thrombosis during aging have been conducted to determine the associated genetic risk factors. Factor V Leiden (rs6025) and the prothrombin G20210A mutation (rs1799963) are the most common prothrombotic variants (incidence of 3–5%) in young and middle-aged populations, and are associated with a three- to seven-fold increase in the risk of venous thrombosis, compared with non-carriers [210,211,212]. The non-O blood group is also an important determinant of venous disease [213,214]. In the young and middle-aged population, blood group non-O is associated with a doubling in the risk of venous thrombosis [215]. In parallel, other studies have shown that, with increased age (> 70 years), the risk of venous thrombosis was 2.2-fold greater in factor V Leiden carriers, 1.4-fold greater in prothrombin G20210A mutation carriers, 1.3-fold greater in those with non-O blood group, and 2.1-fold greater in those with a positive family history of venous thrombosis [216]. The highest risk of venous thrombosis was found in individuals who had both a positive family history and were carriers of one of the two prothrombotic mutations [216]. Yet, genome-wide linkage failed to identify the previously established genetic risk factors for venous thromboembolism, but identified a novel venous thromboembolism susceptibility locus on chromosome 7p [217]. Haplotype tagging SNP *THSD7A* rs2074597 explains part of the chromosomal 7p linkage peak [217]. In addition, an association between venous thromboembolism and genetic variation in HO-1 (*HMOX1*) [183,218], methylenetetrahydrofolate reductase (MTHFR) C677T polymorphism [219], affected homocysteine levels, and plasminogen activator inhibitor-1(PAI-1) 4G/5G mutation [220]. Furthermore, studies have also associated thromboembolism with other pathophysiological conditions, such as inflammatory bowel disease (IBD) and glioma. The genetic factors that have been suggested to interfere in the thrombotic manifestations of IBD include factor V Leiden, prothrombin G20210A, MTHFR 6777T gene mutation, plasminogen activator inhibitor type 1 (PAI-1) gene mutation and factor XIII (val34leu) [221]. Mutant isocitrate dehydrogenase 1 (IDH1) displays potent antithrombotic activity within gliomas, and throughout the peripheral circulation [222].

In Summary, RBC oxidative stress-impaired mechanical properties, deformability and blood rheology, consequently affecting blood flow in the circulation, and this is combined with the compromised phagocytosis of RBC-exposing PS, hemolysis, and genetic polymorphisms and mutations can trigger venous prothrombotic events [223,224], thus predisposing older adults to age-related VT/E in particular, and possibly other cardiovascular diseases in general.

## 7. Conclusions

Oxidative stress is involved in all of the major processes involved in the development of venous thrombosis. RBCs has been demonstrated to be a critical player in hemostasis and thrombosis. RBCs exert significant regulatory effects on blood coagulation, not only through rheology alteration and interactions with differential cells, RBCs themselves, platelets, endothelium and leukocytes, and coagulation factors, but also via RBC MPs generation, and the cross-talk with the complement system. Oxidative stress in RBCs significantly promotes RBC pro-coagulant potential in a variety of ways. ROS in RBCs modify RBCs’ mechanical properties, increase RBC rigidity and RBCs’ interactions with other cells and coagulation factors, and stimulate MPs generation and PS exposure. Further better understanding of the detailed mechanisms by which RBC ROS manipulate the coagulation cascade would provide potential targets for creating novel strategies to prevent or reduce VT/E risk and/or occurrences in mid-life to advanced stage humans, and possibly other disorders with excessive oxidative stress in common.
